# Transition Into LTRC During the COVID-19 Pandemic: An Ethnographic Case Study

**DOI:** 10.1093/geroni/igab046.858

**Published:** 2021-12-17

**Authors:** Megan Davies, Franziska Zúñiga, Hilde Verbeek, Sandra Staudacher-Preite

**Affiliations:** 1 University of Basel, Basel, Basel-Stadt, Switzerland; 2 Maastricht University, Maastricht, Limburg, Netherlands; 3 Nursing Science, Department of Public Health, University of Basel/Basel, Basel-Stadt, Switzerland

## Abstract

COVID-19 has affected long-term residential care (LTRC) disproportionally due to the high-risk population, lack of resources and insufficient preventative measures. Protective measures, including quarantine and strict visitation restrictions have made transitions into LTRC more challenging. Further insight is needed to understand how residents, relatives and staff have experienced this during the COVID-19 pandemic. During four months of fieldwork in a LTRC facility in Switzerland, a rapid ethnography consisting of interviews, observations, informal conversations and document analysis was conducted. This study included a total of 14 residents, 21 healthcare staff from varying departments and 7 relatives of residents. First results indicate that protective measures interfere with a resident’s ability to find meaningful activities and interactions within LTRC as well as the possibility to maintain mobility. This and limited family contact following a move into LTRC prevents a smooth transition from home to LTRC and impacts overall resident quality of life.

